# Long term longitudinal follow-up of an AD-HIES cohort: the impact of early diagnosis and enrollment to IPINet centers on the natural history of Job’s syndrome

**DOI:** 10.1186/s13223-023-00776-5

**Published:** 2023-04-20

**Authors:** Maria Carrabba, Rosa Maria Dellepiane, Manuela Cortesi, Lucia Augusta Baselli, Annarosa Soresina, Emilia Cirillo, Giuliana Giardino, Francesca Conti, Laura Dotta, Andrea Finocchi, Caterina Cancrini, Cinzia Milito, Lucia Pacillo, Bianca Laura Cinicola, Fausto Cossu, Rita Consolini, Davide Montin, Isabella Quinti, Andrea Pession, Giovanna Fabio, Claudio Pignata, Maria Cristina Pietrogrande, Raffaele Badolato

**Affiliations:** 1 Department of Internal Medicine, Fondazione IRCCS Ca’ Granda Ospedale Maggiore Policlinico, Milan, Italy; 2 Department of Pediatrics, Fondazione IRCCS Ca’ Granda Ospedale Maggiore Policlinico, Milan, Italy; 3 Pediatrics Clinic and Institute for Molecular Medicine A. Nocivelli, Department of Clinical and Experimental Sciences, University of Brescia and ASST-Spedali Civili di Brescia, Brescia, Italy; 4 Pediatric Section, Department of Translational Medical Science, Federico II University, Naples, Italy; 5 Pediatric Unit, IRCCS Azienda Ospedaliero-Universitaria di Bologna, Bologna, Italy; 6 Academic Department of Pediatrics (DPUO), Immune and Infectious Diseases Division, Research Unit of Primary Immunodeficiencies, IRCCS, Bambino Gesù Children’s Hospital, Rome, Italy; 7 Chair of Pediatrics, Department of Systems Medicine, University of Rome ‘‘Tor Vergata’’, Rome, Italy; 8 Department of Molecular Medicine, “Sapienza” University of Roma, Rome, Italy; 9 Department of Maternal Infantile and Urological Sciences, “Sapienza” University of Rome, Rome, Italy; 10 Pediatric Clinic, Antonio Cao Hospital, Cagliari, Italy; 11 Section of Pediatrics Immunology and Rheumatology, Department of Pediatrics, University of Pisa, Pisa, Italy; 12 Division of Pediatric Immunology and Rheumatology, Department of Public Health and Pediatrics, “Regina Margherita” Children Hospital, University of Turin, Turin, Italy; 13 Università Degli Studi of Milan, Milan, Italy

**Keywords:** AD-HIES, Job’s syndrome, Immunodeficiency, Inborn errors of immunity, STAT3, Pneumatocele, Staphylococcal infections, Mucocutaneous candidiasis, COVID-19

## Abstract

Job’s syndrome, or autosomal dominant hyperimmunoglobulin E syndrome (AD-HIES, STAT3-Dominant Negative), is a rare inborn error of immunity (IEI) with multi-organ involvement and long-life post-infective damage. Longitudinal registries are of primary importance in improving our knowledge of the natural history and management of these rare disorders. This study aimed to describe the natural history of 30 Italian patients with AD-HIES recorded in the Italian network for primary immunodeficiency (IPINet) registry. This study shows the incidence of manifestations present at the time of diagnosis versus those that arose during follow up at a referral center for IEI. The mean time of diagnostic delay was 13.7 years, while the age of disease onset was < 12 months in 66.7% of patients. Respiratory complications, namely bronchiectasis and pneumatoceles, were present at diagnosis in 46.7% and 43.3% of patients, respectively. Antimicrobial prophylaxis resulted in a decrease in the incidence of pneumonia from 76.7% to 46.7%. At the time of diagnosis, skin involvement was present in 93.3% of the patients, including eczema (80.8%) and abscesses (66.7%). At the time of follow-up, under therapy, the prevalence of complications decreased: eczema and skin abscesses reduced to 63.3% and 56.7%, respectively. Antifungal prophylaxis decreased the incidence of mucocutaneous candidiasis from 70% to 56.7%. During the SARS-CoV-2 pandemic, seven patients developed COVID-19. Survival analyses showed that 27 out of 30 patients survived, while three patients died at ages of 28, 39, and 46 years as a consequence of lung bleeding, lymphoma, and sepsis, respectively. Analysis of a cumulative follow-up period of 278.7 patient-years showed that early diagnosis, adequate management at expertise centers for IEI, prophylactic antibiotics, and antifungal therapy improve outcomes and can positively influence the life expectancy of patients.

**Supplementary Information:**

The online version contains supplementary material available at 10.1186/s13223-023-00776-5.

## Introduction

Autosomal dominant hyper-immunoglobulin E (IgE) syndrome (AD-HIES) is a complex rare inborn errors of immunity (IEI) with an annual incidence of approximately one per million population [1].

AD-HIES STAT3-Dominant Negative (DN) is recognized as a multisystem disorder with both immunologic and non-immunologic features, distinguished by a clinical trial of eczema, recurrent staphylococcal skin and lung infections, and elevated serum IgE levels (above 2000 UI/ml) due to loss-of-function (LOF) mutations of the signal transducer and activator of transcription 3 (STAT3) gene [2].

The disorder was first described by Davis et al. in 1966 and was called Job’s syndrome [3], because the pain and sores of AD-HIES reminded them of the draining skin sores and pustules which the biblical character Job endured (Job 2:7). Six years later, Buckley et al. reported a similar disease with recurrent infections, severe dermatitis, elevated serum IgE levels, and distinctive facial features, and called it as “Buckley syndrome” [4]. Two years later, with the identification of elevated IgE levels, Hill proposed the controversial name “hyper IgE syndrome” (HIES) [5].

Nowadays the term AD-HIES (OMIM #147060) describes patients who present with low levels of inflammatory markers during infections and other multisystem manifestations, other than immunodeficiency.

In 1999, Grimbacher et al. investigated 70 of the relatives of 30 patients with HIES and reported a multisystem disorder with a single-locus AD pattern of inheritance [6]. In 2007, Meneghishi et al. identified signal transducer and activator of transcription-3 (STAT3) as the causative disease gene in AD-HIES due to dominant-negative mutations in the DNA-binding domain [2, 7].

STAT3 protein is a downstream effector of T helper 17 (Th17)-inductive cytokines, including interleukin (IL)-6, IL-12, and IL-23, and is essential for the differentiation of Th17 cells, which are important for eliminating extracellular fungi and bacteria through the production of cytokines, such as IL-17 and IL-22. A mutation in this gene could trigger poor activation of Th17 cells and subsequent defective inflammatory reactions against pathogens [8–10] and a lack of circulating memory B cells [11, 12]. Understanding the molecular pathways of AD-HIES could explain the high incidence of infections caused by *Staphylococcus* and *Candida* species characteristic to this disorder [13]. New insights into the STAT3 pathway can further our understanding for better management of patients with AD-HIES.

Recently, phenocopies of AD-HIES have been reported (autosomal recessive ZNF341 deficiency, partial deficiency of the common receptor chain gp130 encoded by IL6ST, and mutations in ERBB2IP) [14–16].

Based on the extended phenotype of the disease, a scoring system for improving clinical diagnosis, known as the NIH HIES score, has been developed [17]. The modified score developed in 2010 predicts the likelihood of an individual with high serum IgE levels having a mutation in the STAT3 gene [8].

Conventional treatments include antimicrobial prophylaxis and therapies to control the recurrence and severity of infections, while novel treatment strategies, such as monoclonal antibodies targeting allergic manifestations and bone marrow transplantation, are under exploration [1].

Several national or international analyses of patients with AD-HIES investigating molecular and cellular defects have been reported, including 60 French patients [18], 85 patients from the USA [19], two Chinese cohorts of 17 and 20 patients [20, 21], 19 Iranians [22], and 103 patients from India (27 genetically confirmed) [23].

This paper describes the longitudinal and natural history of 30 genetically confirmed Italian patients with AD-HIES STAT3-DN enrolled in the registry of Italian network primary immunodeficiencies (IPINet), providing clinical data that adds to the clinical knowledge in this field. The study highlights the importance of the IEI Registry to longitudinal data collection on AD-HIES and improvement in the condition of patients when cared for in a reference center.

## Patients and methods

### The IPINet Registry

The IPINet Registry, built in 1999 [24], collects all the “historical” patients who have been cared for since 1970 even before their rare disorder had been genetically identified. Patients are entered directly by attending physicians in an online electronic database that runs a dedicated server managed by the Interuniversity Computing Centre (CINECA; https://www.cineca.it/en/progetti/aieop) [25].

All enrolled patients were diagnosed with AD-HIES according to the 2014 protocol by the IPINet group of the Italian Association of Pediatric Haematology Oncology (AIEOP) [26], where definitions, signs, symptoms, diagnostic, and inclusion criteria have been extensively reported. Patients aged > 18 years are considered adults in this study. All patients signed an informed consent form. The local ethics committee approved the registry protocol study.

### Mutation analysis

Patients were analyzed for STAT3 mutations using Sanger sequencing or NGS. Blood samples were sent to the IPINet Reference Lab at the Angelo Nocivelli Institute for Molecular Medicine, Brescia, Italy. One patient was diagnosed at the University of Pavia [27]. Six patients were enrolled at the UCL College of London. These patients contributed to the international cohort that allowed the identification of the causative disease gene STAT3 [8]. All patients provided informed consent for genetic analysis and received genetic counseling.

### Statistical analysis

Clinical features and laboratory data for each patient were collected longitudinally from birth to 2019 (or death). All statistical tests were two-sided and computed using IBM SPSS Statistics 22.0 (IBM, New York, NY).

## Results

### Demographics

In 2019, nine of the 62 IPINet Centers enrolled 30 Italian patients (17 males and 13 females) with AD-HIES, comprising 0.089% of the overall 3352 patients on the IPINet registry, confirming that AD-HIES is a very rare IEI [24]. The cohort also comprises eight “historical” patients who were followed for several years before the registry was started.

Twenty of the Italian patients were adults and ten were children. According to the age of the patients at the last encounter in 2019, the mean age of the cohort was 24.7 years (SD ± 14.2 years; median, 23.6; range, 3.2 to 49.2). The mean age at symptom onset was 12 months (median, 4 months; range, 0–6.1 years), and 66.7% of the patients developed symptoms before the first year of life. The mean age at clinical diagnosis was 16.6 ± 13.9 years (median, 12.1; range, 4 months to 45.8 years).

**Fig. 1:**
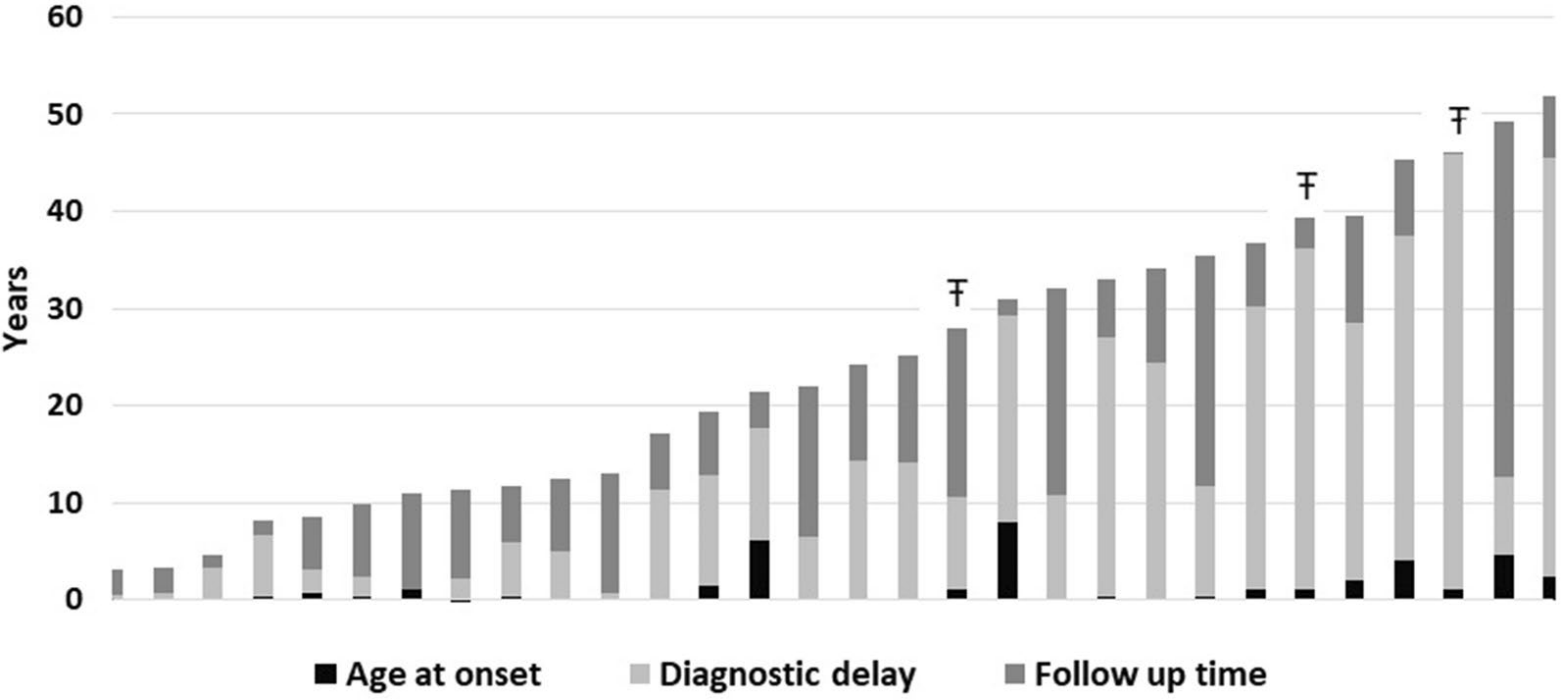
The age of AD-HIES whole cohort at onset, diagnosis and the time of follow up. The bar-plot shows patients’ age in 2019. The three part of the bars are composed by: onset age (black), diagnostic delay time (light grey), follow up time (dark grey). The symbol “Ŧ” identifies the three dead patients

The diagnostic delay was calculated as the time elapsed between the first presenting symptom and the date of either genetic or clinical diagnosis (Fig. 1). If both dates were available, the earliest date was used.

The mean of the time of diagnostic delay was 13.7 ± 13.2 years (median, 10.1; range, 4 months to 44.8 years), with eight patients diagnosed during the third or fourth decade of life (Fig. 1).

The observation time, calculated from the time of onset of symptoms to 2019, was 721.1 years for the whole cohort, with a mean follow-up period after diagnosis of 9.3 ± 7.9 years/patient (follow-up median time, 6.7; range, 2 months to 36.6 years) and a cumulative follow-up period of 278.7 years for all 30 patients.

### Survival

In 2019, 27 patients (90%) were reported to be alive and three deceased (10%). One patient died of massive pulmonary hemorrhage at the age of 28 years due to erosion of the bronchial vein in the context of severe bronchiectasis after 17 years of follow-up. Another patient died at the age of 39 years, only 3 years after AD-HIES diagnosis and arrival at the center, because of the concomitant progression of lymphoma and secondary head-neck cancer. The third patient died of uncontrolled severe sepsis at the age of 46 years with a postmortem diagnosis of AD-HIES, which was confirmed by genetic analysis. AD-HIES was recognized in both of the latter patients after the diagnosis of their children. These patients presented with a “full AD-HIES phenotype”: typical facial features, severe recurrent infections, chronic severe untreatable eczema, and skin abscesses.

### STAT3 mutations

Twenty-nine of the 30 patients underwent genetic analysis of STAT3. The remaining patient died before STAT3 genetic testing became available, but her diagnosis was based on the clinical presentations (typical facial features, high palate, dysodontiasis, recurrent abscesses, recurrent severe pulmonary infections, very large pneumatoceles, necrotizing cellulitis, chronic dermatitis, onychomycosis, and very high serum IgE levels), and an NIH score > 70. Eleven cases (36.7%) were familial (including 5 kindreds), none of which were consanguineous, while the remaining cases were sporadic.

**Fig. 2:**
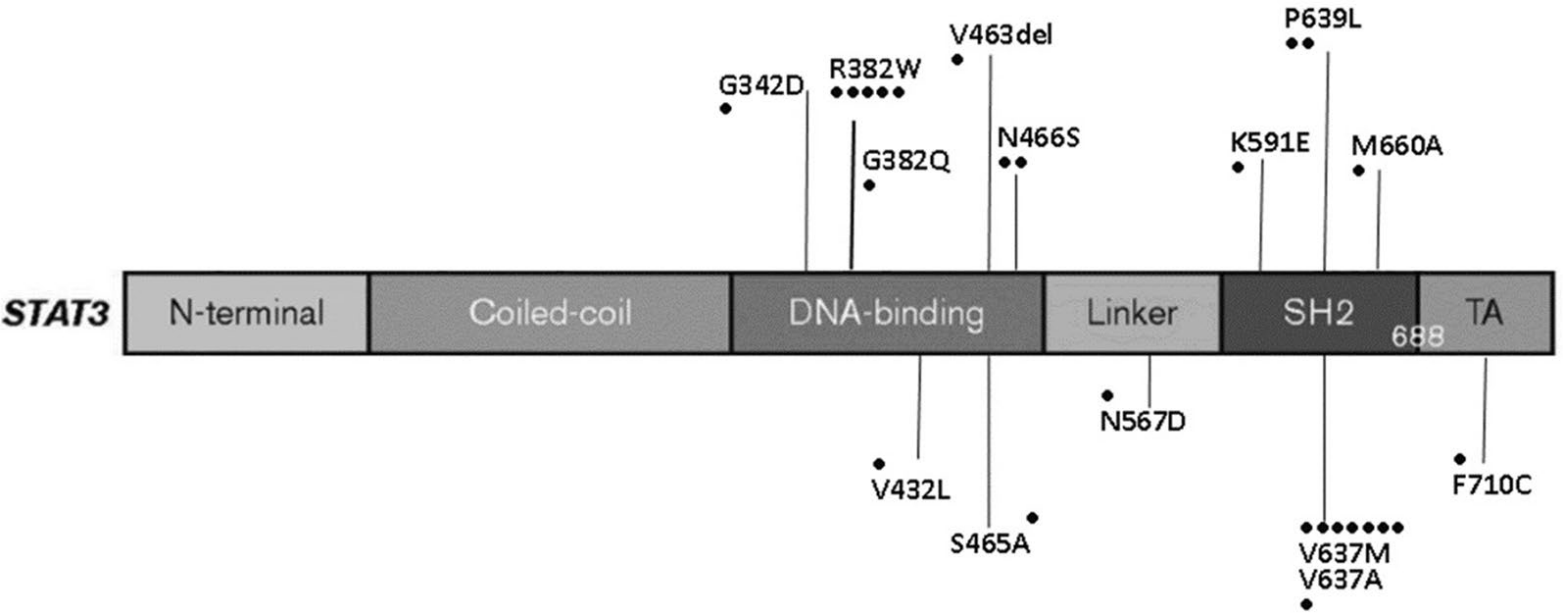
AD-HIES whole cohort: mutations identified on STAT3 gene

The identified mutations were all missense, except for a deletion (V463del) and dominant negative heterozygous LOF mutation of STAT3 [28], which resulted in protein changes, as detailed in Fig. 2 and Table 1. Two previously reported STAT3 mutations (R382W and V637M) were identified in five and seven patients, respectively.

**Table 1:** STAT3 variants identified

STAT3 gene	Mutation		Protein domain	No. patients	STAT3 activity (% of WT) according to
					Asano et al. 2021
exon 10	c.1025 G > A	G342D	DNA-binding	1	< 25%
intron 12	c.1139 + 1G > T	–	DNA-binding	1	0%
exon 13	c.1144 C > T	R382W	DNA-binding	5	0%
exon 13	c.1145 G > A	R382Q	DNA-binding	1	0%
exon 15	c.1294 G > C	V432L	DNA-binding	1	0%
exon 16	c.1387_1389delGTG	V463del	DNA-binding	1	0
exon 16	c.1393 T > G	S465A	DNA-binding	3	0
exon 16	c.1397 A > G	N466S	DNA-binding	2	75–100%
exon 19	c.1699 A > G	N567D	LINKER	1	< 25%
exon 20	c.1771 A > G	K591E	SH2	1	< 25%
exon 21	c.1909 G > A	V637M	SH2	6	< 25%
exon 21	c.1910 T > C	V637A	SH2	1	< 25%
exon 21	c.1916 C > T	P639L	SH2	2	< 25%
exon 21	c.1979 T > C	M660A	SH2	1	–
exon 22	c.2129 T > C	F710C	Transaction	1	0

The STAT3 variants identified in this cohort were previously evaluated by Asano et al. [ref. 28]. They showed that 95.3% of STAT3 variants encoded STAT3 proteins with little or no activity

The P639L STAT3 variant was detected in two patients of the same kindred who presented with the typical AD-HIES clinical phenotype. The father died of non-Hodgkin’s lymphoma three years after his AD-HIES diagnosis. Asano et al. described pathogenic mutations in the same residue (P639) [28].

The M660A STAT3 variation was identified in a patient with newborn skin rash and eczema, infections, and typical facial abnormalities, and known mutations on the same residue (M660) have been reported to be pathogenic [28, 29]. The STAT3 variants identified in this cohort were previously evaluated in terms of biological activity by our group and other authors, which revealed that they act in a dominant-negative fashion (Table 1) [28, 29].

### Laboratory data at diagnosis

At diagnosis, the serum IgE level ranged from 961 to 54,805 kU/mL, with a median level above 5000 kU/mL (mean, 10,253.2 kU/mL; SD ± 13,225.03). The median absolute blood eosinophil count was 438.9 cells/µL with a range from 128 to 18,543 cells/µL (mean, 1537 cells/µL; SD ± 3587.7). The serum IgG level was lower than two standard deviations in two patients with IgG2 deficiency. One patient had selective IgA deficiency.

### Clinical features at onset, diagnosis, and follow-up

#### At disease onset (Fig. 3)

**Fig. 3:**
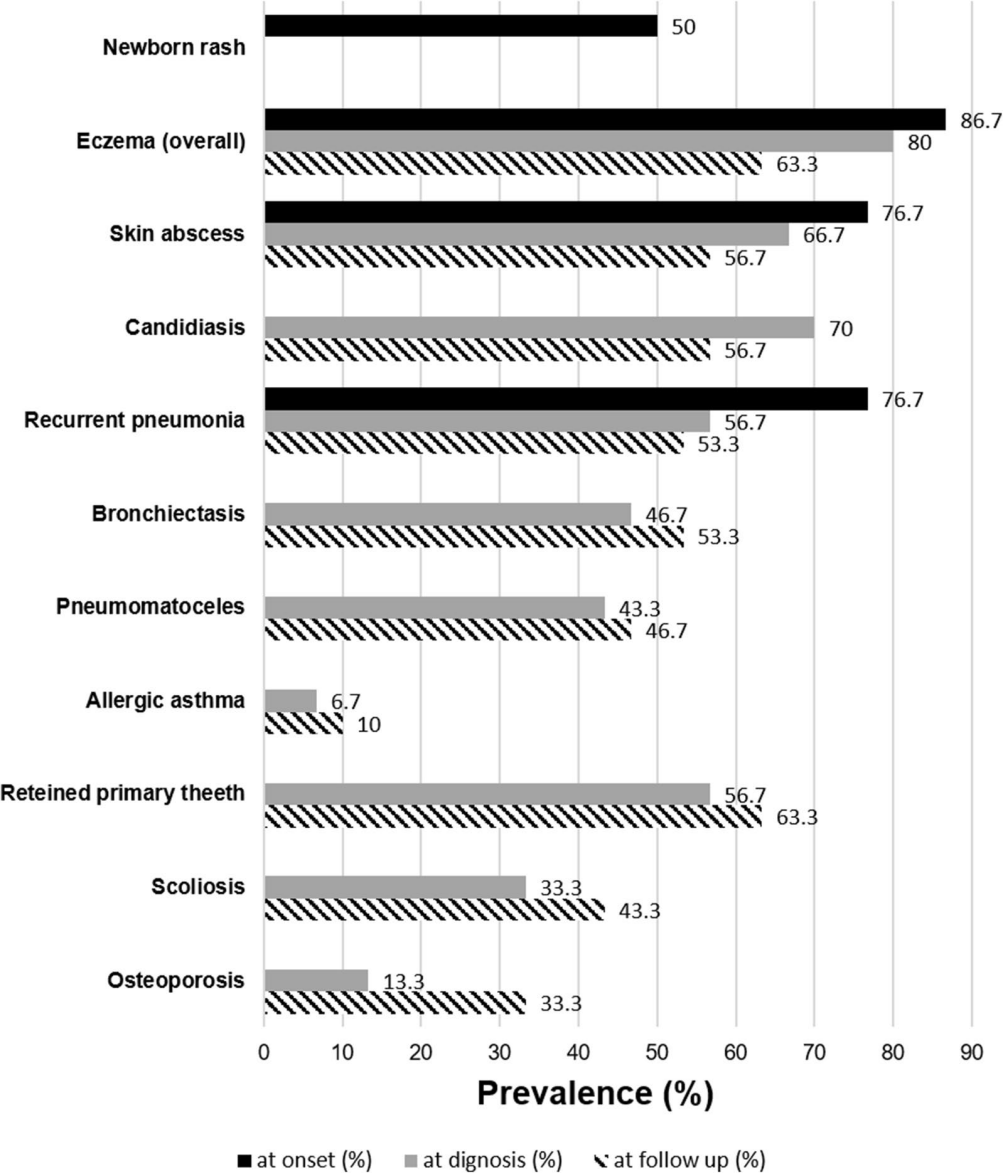
Signs and symptoms at onset, diagnosis and at follow up. The bar-plot shows the prevalence (%) of the signs and symptoms at onset, diagnosis and follow up in the overall cohort

The patient history was remarkable at 93.3% (28/30) for skin involvement. Newborn skin rash was the first clinical sign in 50% (15/30) of the patients, while 61.5% (16/26) experienced severe eczema. At disease onset, 96.7% of patients (29/30) presented with at least one infection, 60% of which occurred before 30 months of life.Recurrent otitis and sinusitis were present in 46.7% (14/30) and 30% (9/30) of patients, respectively. Osteomyelitis (2/30) and sepsis (2/30) were both present at disease onset in 6.7% of the patients.

#### At disease diagnosis (Fig. 3)

Persistent eczema was reported in 80.8% (21/26) of the patients at diagnosis. Of these, 66.7% (20/30) had a medical history of skin abscesses. Pyodermitis was reported at diagnosis in 33.3% of the patients (10/30). Post-infective pneumatoceles were observed in 43.3% (13/30) and bronchiectasis in 46.7% (14/30) of the patients due to severe pulmonary involvement. Invasive pulmonary fungal infection was found in 13.3% (4/30) of the patients. Before diagnosis, a patient had a pulmonary *Staphylococcus* abscess that required lobectomy at 1 year of age, another had a kidney abscess at 8 years of age, and a third had a rectal abscess during the neonatal period.

The “characteristic face” was the most commonly observed skeletal sign, recognized in 83.3% of the patients. Retained primary teeth were the second most frequently observed (56.7%, 17/30), while a high palate was found in 50% (15/30) of the patients.

Scoliosis was diagnosed in 33.3% (10/30) of the patients. Hyper-extensive joints were recognized in 30% (9/30), and another 30% (9/30) experienced bone fractures (only two patients had both hyper-extensive joints and fractures). Osteoporosis was identified in four adults at the time of AD-HIES diagnosis.

Allergic manifestations were reported in several patients: two (6.7%) presented with food allergies and two (6.7%) had allergic asthma. One patient had a history of anaphylactic shock (patient with a STAT3 mutation in the linker domain) [27]. Under suspicion of allergic manifestations, 13 patients (43.3%) underwent both blood allergen-specific IgE and cutaneous prick tests. The RAST results were positive in 11 patients, ranging between 30 and 100% for the allergens tested (six patients > 90%). The prick tests of the corresponding allergens were positive in only four patients: three patients at 10% and one at 40%.

#### During follow up (clinical course, treatments, and procedures) (Fig. 3)

At the latest follow-up, the prevalence of infective airway involvement was 56.7%. All patients were treated for acute infections or complications based on microbiological analyses with the appropriate use of antibiotics or antifungal drugs as recommended [26].

After diagnosis, considering the recurrent infections characteristic to this disease, chronic prophylaxis (local or systemic) was started in 70% (21/30) of patients. Antibiotic prophylaxis was administered to 21 patients, including 18 patients treated with trimethoprim-sulfamethoxazole and 2 with amoxicillin-clavulanate. Azithromycin was administered to the remaining patient for bronchiectasis prophylaxis. (see Additional file 1: Table S1).

During the overall follow-up, the number of patients with localized skin abscesses decreased from 76.7% to 56.7% (Fig. 3), and the rate of patients with pneumonia decreased from 76.7% to 46.7%, especially if the patient showed good compliance with long-life therapy and regular follow-up. At the latest follow-up, two more patients had post-infective bronchiectasis and one more patient developed pneumatoceles (Fig. 3) despite antibiotic prophylaxis. Pulmonary hemorrhaging occurred in two patients with severe post-infective parenchymal lung disease, which was lethal in one of the patients.

Pulmonary infection by *Aspergillus* *fumigatus* occurred after diagnosis in four patients, one of which had aspergilloma.

Antifungal prophylaxis was started in 12 patients because of recurrent invasive fungal infections (*Aspergillus* *fumigatus*); six were treated with fluconazole, four with itraconazole, and the remaining two with voriconazole.

Chronic local therapies were initiated in 15 patients for eczema, while the others were treated only on demand.

The number of patients with lifelong severe and very itchy eczema requiring chronic antihistamine therapy decreased from 16 to 8 during follow-up. The occurrence of mucocutaneous candidiasis and onychomycosis decreased during follow-up from 70 to 56.7% (17/30) and 56.7 to 40% (12/30), respectively.

Several episodes of deep infections were observed during the follow-up period; one patient had a recurrent prostatic abscess associated with recurrent bacterial cholangitis and osteomyelitis. Another patient had liver and pancreatic abscesses. Two female patients had recurrent breast abscesses, and one patient had recurrent mastoiditis.

Dental infections and complications were common in this cohort and resulted in severe outcomes, as previously reported [30]. Signs of skeletal involvement, such as the typical facial features, were recognized in 86.7% (26/30) of the patients, scoliosis was diagnosed in 43.3%, and hyper extensive joints were observed after adolescence. Among the adult patients, 50% (10/20) developed osteoporosis (identified by DXA), which became clinically evident after 30 years of age.

The registry did not collect specific data regarding fertility. However, we registered seven adult patients, three females and four males, with nine children among them, six of which were affected, and three were healthy.

Pregnancy was prospectively observed. One patient that experienced recurrent breast abscesses also experienced them during pregnancy. After vaginal delivery, breastfeeding was interrupted owing to relapsing breast abscesses. After 6 months, the patient developed breast cancer and was treated with mastectomy and hormonal therapy.

Malignancy was diagnosed in 13.3% (4/30) of patients. Two patients developed non-Hodgkin’s lymphoma, which was diagnosed at the time of diagnosis. One patient had breast cancer (as mentioned above). One patient had a neuroendocrine neoplasm of the stomach mucosa.

The pneumococcal vaccine was administered after diagnosis in 8/30 (26.7%) patients, including two adults, without any adverse events. Three patients with absent antibody responses to one or more vaccinations and one with a low IgG2 titer were started on immunoglobulin replacement therapy.

Many patients reported surgical procedures, including surgical tooth extractions, drainage of skin abscesses or other sites, and biopsies of the gastrointestinal tract, lymph nodes, liver, and lungs. A major surgical procedure observed in four patients was lobectomy, including two patients who underwent lobectomy twice. The indications for lobectomy were the diagnosis of lung abscesses in two patients and large pneumatoceles in the other two, including one that was complicated by aspergilloma and was previously reported [31].

In the years after lobectomy, one patient underwent hemicolectomy for abscesses and another one underwent cholecystectomy and prostatectomy because of chronic cholangitis and recurrent prostatitis. One patient underwent a mastoidectomy. Another patient underwent nephrectomy during childhood for a kidney abscess, was found to have kidney failure at the time of diagnosis at the age of 20 years, and required chronic hemodialysis 2 years later.

### COVID-19 disease

During the redaction of this paper, the SARS-CoV-2 infection caused the coronavirus disease 2019 (COVID-19), and the pandemic occurred and had a great impact in Italy.

Among the 27 patients that were alive, two children and five adult patients (aged < 40 years) were infected with SARS-CoV-2. The two children were not previously vaccinated; one contracted COVID-19 in November 2021 and was treated with the monoclonal antibody banlavimib + etesevimab and became negative after 28 days; the second was infected in January 2022 and was completely asymptomatic and negative after ten days without any specific treatment.

Of the five adults who contracted COVID-19, one was infected October 2020 and did not require hospital admission or treatment; another who was infected in March 2021 required ventilation in the intensive care unit because of underlying severe chronic parenchymal lung damage. He received remdesevir and two-dose hyperimmune plasma, which resulted in slow improvement. He tested negative after 12 weeks, but he developed several multidrug-resistant bacterial lung infections and was only discharged after 14 weeks, with oxygen therapy to be continued at home. Three more adults were infected in January 2022. All patients had mild disease; two were treated with sotrovimab and one with casirivimab + imdevimab. They recovered within one week. The adult patients were vaccinated against-SARS-CoV-2 with BNT162b2, all without complications and with antibody responses similar to those reported for healthy subjects [unpublished data].

## Discussion

This manuscript serves as a look at the natural history of 30 patients with confirmed AD-HIES STAT3 negative-dominant mutations in an Italian Registry with a cumulative observational time of 721.1 patient-years (since the onset of the disease). It expands on the STAT3-data presented in a previous publication which looks at the whole group of patients with HIES from the IPINet Registry [32]. This study documents a cumulative follow-up period of 278.7 patient-years after diagnosis which is one of the longest reported to date [18, 19], allowing us to describe and better understand the natural history of this intriguing and rare disease.

Currently, 150 STAT3 variants have been reported in patients with HIES. In one of our previous studies, we functionally characterized several STAT3 mutations identified in this cohort [29]. In patients with mutations in the SH2 domain (V637M), we observed a marked reduction in STAT3 phosphorylation as compared to healthy controls, and abnormal DNA-binding activity. We observed a profound cytokine imbalance, particularly in the impairment of IL-10 signaling. In fact, the expression of anti-inflammatory molecules such as SOCS3, IL-1ra, and CXCL8 was reduced, and abnormal IL-10-derived dendritic cell maturation may be the reason for the increased production of pro-inflammatory cytokines. A comprehensive study by Asano et al. (2021) genetically characterized STAT3 variants in HIES. It included all variants found in the patients of our cohort. They established that the pathogenic mechanisms of heterozygous AD-HIES STAT3 mutations may rely only on negative dominance [28]. They showed that 95.3% of STAT3 variants encoded STAT3 proteins with little or no activity.

The data in this study are presented by analyzing incidence of manifestations at the time of diagnosis versus follow-up, showing improved outcomes after the intervention of an IEI expert.

Despite the characteristic clinical features being present since a very young age (67.7% of patients experienced disease onset before the age of 12 months), the median age at diagnosis was 12.1 years, similar to the USIDNET cohort (13.8 years) [19], and approximately double that of both the French (6.8 years) [18] and the Chinese cohorts (5.8 years) [20]. The diagnostic delay found in the present study was 16.6 years ± 13.9, which is consistent with the fact that most of the patients that came to at the IEI referral centers were already in adulthood.

In this cohort, two fathers were diagnosed only after their sons were referred to the referral IEI center. Although both presented typical phenotypes, had many infections, and underwent many medical evaluations, diagnosis was previously not performed, and they died within a short time after arriving at the IEI center. This is frustrating because the first patient in this cohort was diagnosed at 10 years old in one of the IPINet centers in 1977, a few years after the first case report, and the diagnosis was based on her typical clinical AD-HIES features, even before the identification of the causative disease gene STAT3.

**Fig. 4:**
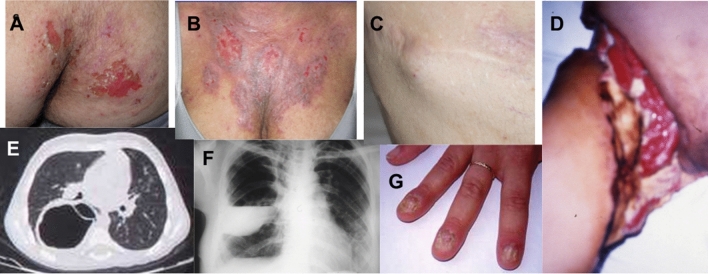
Pathognomonic signs. The figure shows the characteristic events of the patients with AD-HIES: **A** and **B** chronic eczema; **C** cold abscess of chest skin; **D** right leg with necrotizing cellulitis after liposuction surgery; **E** pneumatocele with aspergilloma; **F** pneumatocele with empiema; **G** onychomycosis;

However, general practitioners, pediatricians, pulmonologists, and dermatologists who are not aware of primary immunodeficiencies often still do not recognize the pathognomonic signs of AD-HIES (Fig. 4) and often misdiagnose elevated IgE and eczema as signs of allergy or chronic urticaria, even if there is a remarkable medical history for recurrent infections, even as severe as skin abscesses and pneumatoceles (Fig. 3).

In patients with AD-HIES, despite RAST results being positive for most of the allergens tested, prick tests are often negative and do not confirm allergies for food and inhalants. Indeed, it has been demonstrated that IgE, even though produced in higher quantities, has a lower affinity for allergens [33]. The finding of elevated serum IgE and negative prick test results cast doubt on the diagnosis of allergies. This is a clue for AD-HIES: a high discrepancy should be considered in the differential diagnosis.

**Fig. 5:**
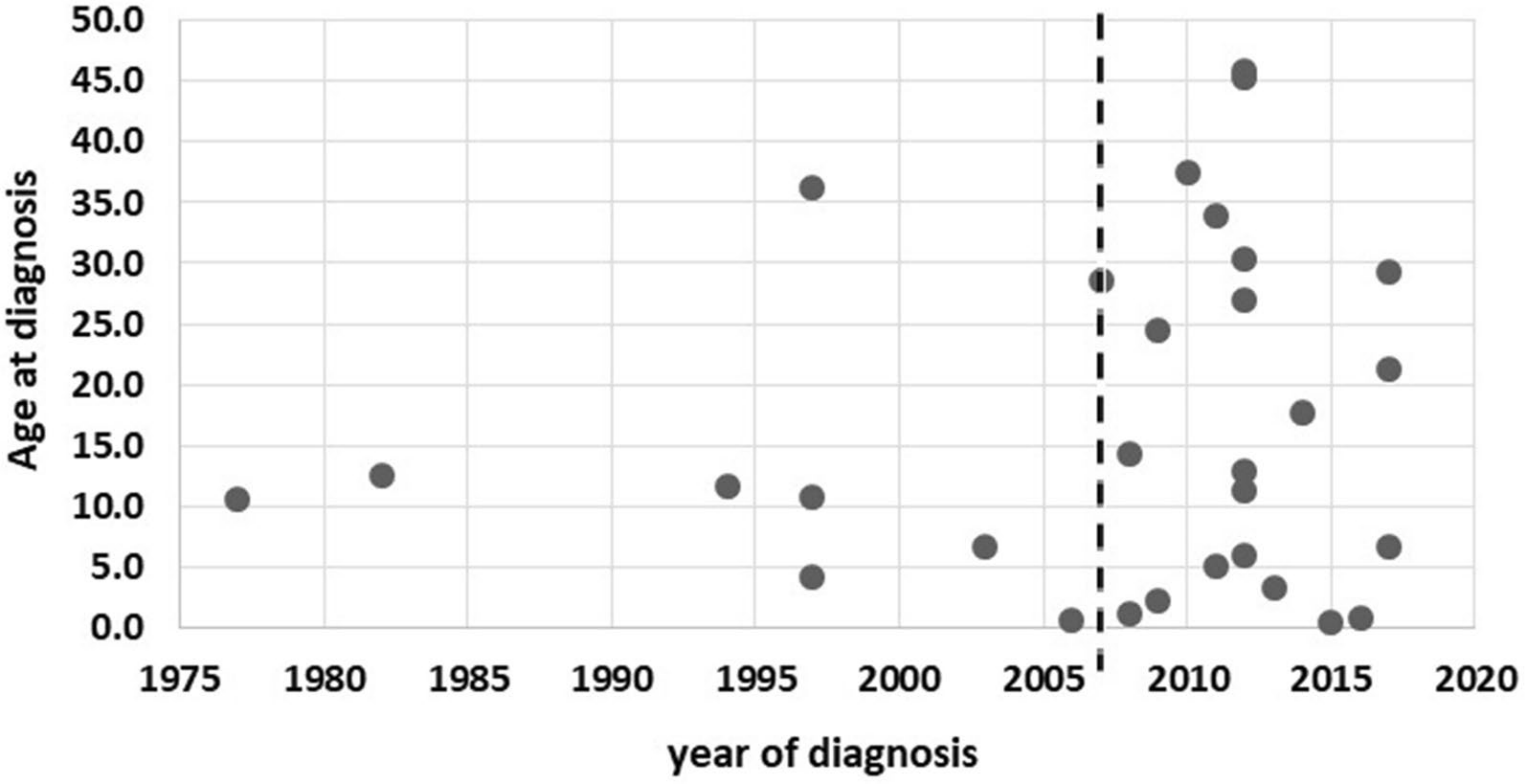
Distribution of diagnosis according to the patient’s year of diagnosis and his/her age at diagnosis. The plot shows patients’ age at diagnosis related to year of diagnosis. In 2007 the STAT3 gene was identified

As shown in Fig. 5, analysis of the diagnostic delay in our cohort revealed an improvement in the number of diagnoses after 2007 where the gene responsible for causing AD-HIES was identified, especially among adults who were not clinically diagnosed in childhood. Understanding the genetics behind the disease also improved diagnosis before the age of 5 years in many children, reducing diagnostic delay.

The patients’ clinical pictures and findings in this study resemble those of other reported AD-HIES cohorts [18–23]. However, we observed considerable clinical variability across individuals bearing the same heterozygous STAT3 mutation, including between those from the same kindred. Newborn rash, which is considered pathognomonic, was observed in 50% of the cases, as in other cohorts [18, 34]. As described elsewhere [18, 20, 22], the most frequent signs at onset were eczema (86.7%) and infections (76.7%) such as recurrent skin abscesses, pneumonia, and mucocandidiasis.

Lung disease and its complications are reported to be the main features affecting the prognosis of these patients. Similar to the USIDNET cohort [19], 76.7% of our patients had at least one historical occurrence of pneumonia reported at diagnosis. The rate of patients with bronchiectasis (46.7%) or pneumatoceles (43.3%) at diagnosis was higher than that of the USIDNET cohort [19], but not significantly different from that of the French cohort [18].

Longitudinal data showed that the rate of patients who had at least one pneumonia occurrence during the follow-up period decreased from 76.7% to 46.7% due to antimicrobial and antifungal prophylaxis.

Regardless of this, however, lung damage progressed (bronchiectasis + 6.6% and pneumatoceles + 3.3%), and four patients required lobectomies to treat the mucoceles. Long diagnostic delay also contributed to lung damage and poor pulmonary tissue repair due to the STAT3-LOF mutations [35].

Current recommendations for the treatment of AD-HIES are largely supportive, and include continuous prophylactic antibiotics, antifungal coverage, and early treatment of infections. Among the overall cohort, 70% of patients were started on antimicrobial prophylaxis at the time of diagnosis, which is comparable to that of the USIDNET cohort [19]. Among the treated patients, 95% were treated with trimethoprim-sulfamethoxazole or equivalent, and 57.1% with antifungal prophylaxis.

We observed that the number of patients receiving antifungal prophylaxis increased over time as the rate of patients that were switched to itraconazole and voriconazole from fluconazole increased owing to the resistance of *Aspergillus* and *Candida spp*. Topical therapy for eczema, in combination with antibiotic prophylaxis, reduced the rate of patients with both severe eczema (− 19.4%) and skin abscesses (− 20%).

STAT3 plays a role in B-cell function, with both a reduction in antibody production and interaction with follicular T helper cells. Particular attention should be given to the evaluation of the IgG subclass serum levels, the switched B-cell memory percentage, and the response to pneumococcal vaccinations at diagnosis (and during follow-up). Despite the high rate of pulmonary damage, only eight patients received a pneumococcal vaccine. In a recent report by USIDNET [19], pneumococcal vaccinations exposed patients to the risk of skin ulceration. We did not observe such adverse events in our patients, neither in the children, nor in the adults who underwent pneumococcal vaccination (both polysaccharide or conjugated). In our opinion and experience, pneumococcal vaccines should be highly recommended for both adults and children. Further study is necessary to confirm the effects of these vaccines.

During the COVID-19 pandemic, vaccination with mRNA vaccines was extensively performed on our cohort without any complications, and normal antibody responses were observed.

Three patients were started on immunoglobulin replacement therapy (Ig RT) because of a lack of response to vaccination and low serum IgG2 levels. Previous reports by both the USIDNET and French cohorts revealed that the use of Ig RT in patients with AD-HIES can reduce the number of bacterial pneumonia cases [18, 19], as we observed. However, there are still no studies that address the efficacy of the regular use of Ig RT in patients with AD-HIES, and whether its safety should be trusted in patients with hyper-IgG serum levels, which were encountered in four adults with AD-HIES in this cohort.

None of the patients in this cohort had underwent marrow transplantation. There are still many controversies regarding this treatment for AD-HIES [36, 37], and further data concerning the comprehensiveness of the disease are needed.

Patient survival seems to be affected mainly by permanent and irreversible complications related to several infections that occurred before diagnosis. In many patients, the correct diagnosis was made, and therapy started when the patient was referred to an IEI expert. However, patients often arrive at the IEI centers in adulthood and already have many permanent and irreversible complications.

The observed incidence of age-related complications, such as osteoporosis and malignancies, was higher with an increase in patient age and may be related to the underlying genetic defect. Osteoporosis should be evaluated from childhood, and adequate preventive therapy should be implemented.

Adherence to therapy and follow-up is an important factor in avoiding complications and improving life expectancy, particularly in the transition stage from pediatric to adult IEI centers. A tailored tool for quality-of-life assessment would help improve the management and outcomes of patients during this transition period.

In conclusion, through the longitudinal analysis from diagnosis to follow-up, this study highlights some pivotal points for the rare disorder AD-HIES that we would like to propose: diagnostic delay should be avoided; improving the knowledge in non-IEI specialists about the clinical trial of AD-HIES and its red flags should be a priority; medical history should be carefully evaluated and if AD-HIES is suspected, genetics should be always addressed; at diagnosis, all patients with AD-HIES should be screened and treated for any complications, in particular for pulmonary complications; both antibiotic and antifungal prophylaxis plus local therapy for chronic eczema and mucocandidiasis prevention should be started; vaccinations should be proposed as current pneumococcal vaccinations seem to be safe in patients with HIES; B-cell defects should be ruled out and patients should be treated in the presence of hypogammaglobulinemia; osteoporosis should be evaluated and prevented or treated at any age; and adult female patients should be carefully screened for breast abscesses, as well as adult males for prostate abscesses.

The extremely complex medical history of some of these patients prompted this manuscript, as did the intent to contribute to setting cornerstones for a shared international protocol to manage and treat this rare disease. This was done with the aim of improving the suffering of these patients who often endure everything with the proverbial “patience of Job”.

## Supplementary Information

**Additional file 1 - Table S1.** The table details findings (signs and symptoms) at onset, diagnosis, overall follow up and latest follow up.

## Data Availability

Data are archived online in the AIEOP-IPINet electronic Registry at http://www.aieop.org/areariservata/?pagina=inputdati. IPINet Registry data privacy regulations allow the use of repositories only by researchers of IPINet centers, as covered by ethical approval and patient consent. The datasets analyzed during the current study are available from the corresponding author upon reasonable request.
